# Explainable Machine Learning Model to Prediction EGFR Mutation in Lung Cancer

**DOI:** 10.3389/fonc.2022.924144

**Published:** 2022-06-23

**Authors:** Ruiyuan Yang, Xingyu Xiong, Haoyu Wang, Weimin Li

**Affiliations:** ^1^ Department of Respiratory and Critical Care Medicine, West China Hospital, Sichuan University, Chengdu, China; ^2^ Institute of Respiratory Health Frontiers Science Center for Disease-related Molecular Network, West China Hospital, Sichuan University, Chengdu, China; ^3^ Precision Medicine Center, Precision Medicine Key Laboratory of Sichuan Province, West China Hospital, Sichuan University, Chengdu, China; ^4^ The Research Units of West China, Chinses Academy of Medical Sciences, West China Hospital, Chengdu, China

**Keywords:** EGFR mutation, lung cancer, prediction, machine learning, SHAP value

## Abstract

**Objectives:**

The aim of this study is to determine whether the clinical features including blood markers can establish an explainable machine learning model to predict epidermal growth factor receptor (EGFR) mutation in lung cancer.

**Methods:**

We retrospectively analyzed 7,413 patients with lung adenocarcinoma (LA) diagnosed by gene sequencing in West China Hospital of the Sichuan University from April 2015 to June 2019. The machine learning algorithms (MLAs) included logistic regression (LR), random forest (RF), LightGBM, support vector machine (SVM), multi-layer perceptron (MLP), extreme gradient boosting (XGBoost), and decision tree (DT). Demographic characteristics, personal history, and blood markers were taken into. The area under the receiver operating characteristic curve (AUC) and SHapley Additive exPlanation (SHAP) value were used to explain the prediction models.

**Results:**

Of the 7,413 patients with LA (47.6%), 3,527 were identified with EGFR mutation; RF achieved greatest performance in predicting EGFR mutation AUC [0.771, 95% confidence interval (CI): 0.770, 0.772], which was like XGBoost with AUC (0.740, 95% CI: 0.739, 0.741). The five most influential features were smoking consumption, sex, cholesterol, age, and albumin globulin ratio. The SHAP summary and dependence plot have been used to explain the affection of the 12 features to this model and how a single feature influences the output, respectively.

**Conclusion:**

We established EGFR mutation prediction models by MLAs and revealed that the RF was preferred, AUC (0.771, 95% CI: 0.770, 0.772), which was better than the traditional models. Therefore, the artificial intelligence–based MLA predicting model may become a practical tool to guide in diagnosis and therapy of LA.

## Introduction

Lung cancer has become a commonly diagnosed tumor, which accounts for approximately 11.4% of all cancers diagnosed and 18% of cancer-related death (1–4), which induced a high economic burden and life loss. The first choice is still surgical resection when lung cancer occurs and stage II or III patients also receive adjuvant therapy followed by surgery (5, 6) for decreasing the possibility of progression and relapse and further increased the progression-free survival (7, 8). However, many patients have missed the chance of surgical therapy with a visit to doctors because of the symptoms. They usually cannot access surgery; usually, chemotherapy or radiotherapy is preferred for this part of unresectable or inoperable patients (7). The traditional plan is Cisplatin-based adjuvant chemotherapy. With the rapidly evolving treatment landscape, tyrosine kinase inhibitors (TKIs) have an expanding place in lung cancer therapy (6), as first-line or adjuvant treatment plans. Patients get greater clinical benefit compared with traditional chemotherapy, for those who were confirmed with targetable gene mutations, like Epidermal growth factor receptor (EGFR), anaplastic lymphoma kinase (ALK) (7, 8).

EGFR, a tyrosine kinase receptor, has a mutation rate of about 10% in lung adenocarcinoma (LA) and an unignorable higher rate in non-smoking patients. It is the earliest gene to be uncovered, and EGFR-TKIs were adopted for clinical work, which markedly changed the therapy strategy and yielded better therapeutic prospects in lung cancer, particularly in adenocarcinoma (9, 10). The diagnosis still depends on tumor tissue biopsy (11), through broncho fiberscope, puncture biopsy, or surgery, which have significant risks, such as surgery-related and time costs. Hence, non-invasive and fast method to confirm EGFR mutation is needed in clinical work. From logistic regression (LR) to machine learning, different methods were adopted to evaluation genotype mutation with AUC ranging from 0.65 to 0.75. Recent studies made use of CT or PET-CT images, and the sensitivity was increased to 0.81 (12).

In the study, an explainable model finds the significant influential factors for EGFR mutation with SHAP value. We utilized retrospective data including demographic characteristics and clinical examination in a large sample of patients with LA and finally selected the model with the best performance. It is expected that this type of model would be used for reference by clinicians.

## Materials and Methods

### Study Participants

We retrospectively collected the clinical records of 7,413 patients who underwent LA diagnosis in West China Hospital of the Sichuan University, from April 2015 to June 2019.

### Statistical Methods

Continuous data are presented as the mean ± standard deviation (SD) or median (Q1, Q3), and categorical data are described as numbers (%). Student’s t-test or one-way ANOVA (analysis of variance) was used for normally distributed continuous variables. The Newman–Keuls or Student–Newman–Keuls method was used for multiple samples. The Kruskal–Wallis test was used for the non-normally distributed continuous variables. The Chi-square test and the Fisher discriminant analysis were used to evaluate the difference in categorical variables such as sex and smoking status. We assessed the predictive performance according to the AUC, to evaluate the prediction and accuracy of various machine learning models abovementioned in the test set, and bootstrap methods with 1,000 bootstrap replicates were used to derive 95% confidence interval (CI).

### Machine Learning Models

We used the following supervised machine learning methods to develop the predictive models, which were novel and traditional machine learning methods used for the problem of classification: LR, random forest (RF), LightGBM, support vector machine (SVM), multi-layer perceptron (MLP), extreme gradient boosting (XGBoost), and decision tree. LR is a simple and more efficient method for binary and linear classification problems. It is a classification model, which is very easy to realize and achieve satisfied performance with linearly separable classes. It is an extensively employed algorithm for classification in medical study. Multilayer perception, known as artificial neural network (ANN), is a model that is inspired by the human brain and the way it functions. A standard ANN has an input layer, an output layer, and at least one hidden layer between input and output. ANN always has several layers of nodes, definite link patterns and layer connections, connection weights, and node (neuron) and activation functions that map weighted inputs to outputs. Throughout the training process, the weights are changed. The backpropagation algorithm is a technique to train ANNs, and it has the following two key stages: propagation and weight update. SVMs are supervised learning models with associated learning algorithms that analyze data for classification and regression analysis. It performs well in high-dimensional spaces and is still effective in cases where the number of dimensions is greater than the number of samples. In other hand, it uses a subset of training points in the decision function (called support vectors), so it is also memory-efficient. Decision tree is a non-parametric supervised learning method used for classification and regression. The goal is to create a model that predicts the value of a target variable by learning simple decision rules inferred from the data features. In decision analysis, a decision tree can be visually and explicitly used to represent decisions and decision-making. Furthermore, decision tree is the basis of the following three models: RF, XGBoost, and LightGBM. RF consists of many random decision trees. It uses random sample of original data and random subset features to build the model. Each tree gives a classification, and we say the tree “votes” for that class. The forest chooses the classification having the most votes (over all the trees in the forest). XGBoost provides a parallel tree boosting that solve many data science problems in a fast and accurate way. Three main forms of gradient boosting are supported: gradient boosting algorithm, stochastic gradient boosting, and regularized gradient boosting. In the end, LightGBM also uses tree-based learning algorithms. It can be used in classification, regression, and many more machine learning tasks. It is designed to be distributed and efficient with the following advantages: faster training speed and higher efficiency, support of parallel learning, and capable of handling large-scale data. All data of 7,413 patients were included, in which, among the seven models, the best model to predict EGFR mutation is found (13–15).

## Results

Of the 30,052 patients with lung cancer, we gathered the clinical statistics of 7,413 patients with LA who meet the inclusion criteria from April 2015 to June 2019 in West China Hospital. All 7,413 patients with LA are confirmed by gene examination. Among them, 3,527 patients (47.6%) were diagnosed with EGFR mutation, and the remaining 3,886 patients were negative ones, in which the incidence was consistent with previous studies in Asia (16). Moreover, the mean age was 56.93 years. EGFR mutation cases were typically women, with less exposure to smoke and drink compared with controls (all P < 0.001). Ninety-one demographics and blood markers were included, and all features were from the first visit. In addition, nearly all variables, the differences between the train and test sets, were non-significant. Because of a large number of parameters included, we only show parameters included in our final model in **Table 1**. The full information could be found in **Table S1**.

**Table 1:** Patient characteristics and blood markers.

Variables	EGFR–Wild Type	EGFR-Mutation	P-Value
Patient population, n (n%)	3,886	3,527	
**Demographic data**			
Gender, n (%)			<0.001
Female	1,446 (37.210)	1,970 (55.855)	
Male	2,440 (62.790)	1,557 (44.145)	
Smoking Consumption, n (%)			<0.001
No	1,814 (46.873)	2,490 (71.001)	
Yes	2,056 (53.127)	1,017 (28.999)	
Age (year), mean (SD)	56.965 (10.749)	56.893 (10.197)	0.768
**Blood routine**			
Hemoglobin (g/L), mean (SD)	120.959 (17.833)	122.760 (17.470)	<0.001
Platelet(10^9^/L), mean (SD)	215.069 (83.880)	211.581 (81.704)	0.079
Neutrophils%, mean (SD)	64.071 (12.673)	63.993 (11.888)	0.794
Lymphocyte%, mean (SD)	24.315 (10.758)	24.825 (10.108)	0.043
**Blood biochemistry**			
Cholesterol (mmol/L), mean (SD)	4.773 (1.006)	4.726 (1.002)	0.053
Albumin Globulin Ratio, mean (SD)	1. 527 (0.329)	1.587 (0.320)	<0.001
Glutamyl Transpeptidase (IU/L), mean (SD)	36.925 (22.541)	33.808 (22.600)	<0.001
Aspartate Aminotransferase (IU/L), mean (SD)	24.854 (8.265)	24.952 (8.444)	0.623
**Tumor markers**			
Carcinoembryonic Antigen (ng/ml), median [Q1, Q3]	6.120 [2.440, 25.348]	6.640 [2.300, 30.797]	0.881

**Figure 1:**
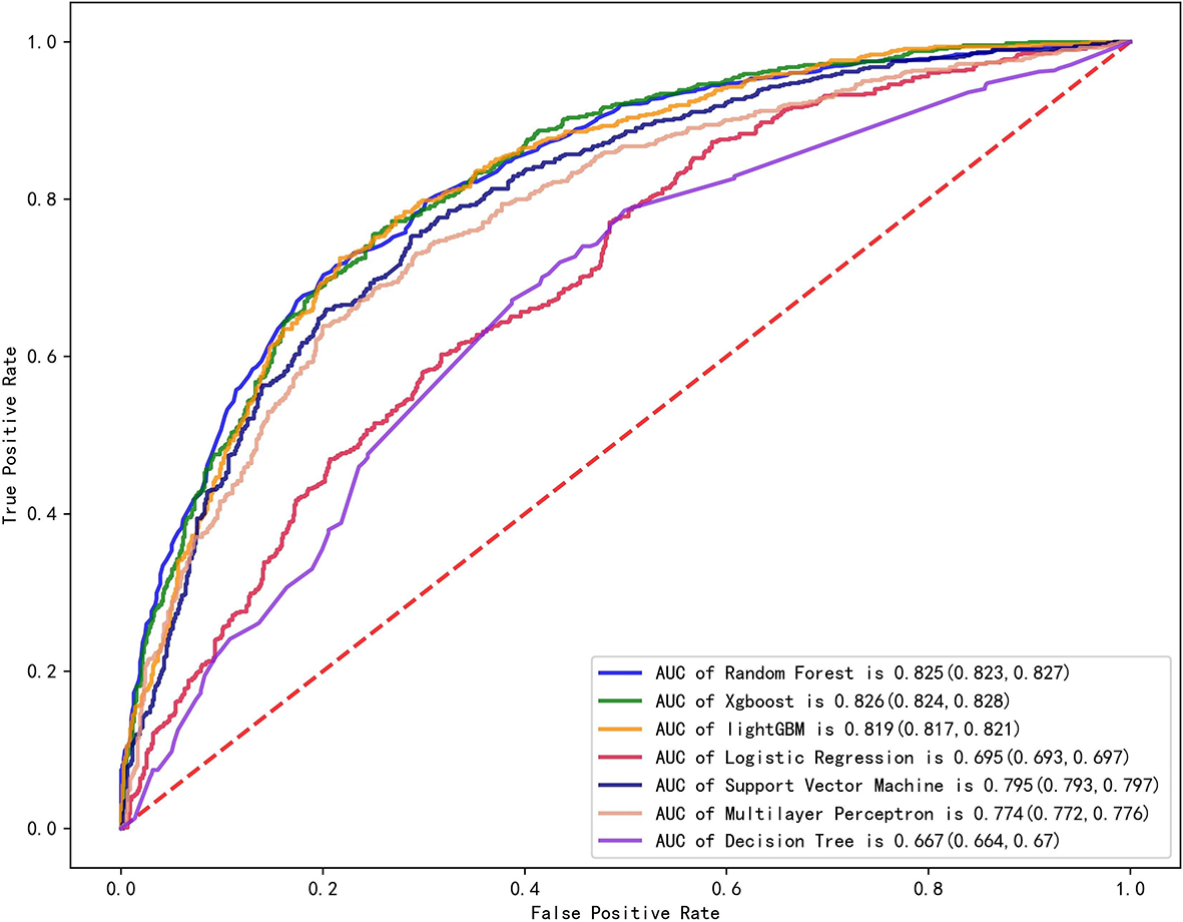
Comparison of AUCs among seven machine learning models with ROC; RF got the greatest AUC for single model prediction.

We established final models with 12 features elected by the least absolute shrinkage and selection operator (LASSO) algorithm and clinical experience using machine learning algorithms (MLAs) above, including smoking consumption, sex, cholesterol, age, albumin globulin ratio, glutamyl transpeptidase, hemoglobin, carcinoembryonic antigen (CEA), platelet, neutrophils, lymphocytes, and aspartate aminotransferase. In the test set, RF achieved great performance in terms of predicting EGFR mutation with AUC (0.771, 95% CI: 0.770, 0.772), which was similar to XGBoost with AUC (0.740, 95% CI: 0.739, 0.741). The quantitative performance and the ROC curves had been established on **Table 2** and **Figure 1**.

**Table 2:** The quantitative performance and the ROC curves of included models.

Model	AUC	Youden_Index	Sensitivity	Specificity
RF	0.825 (0.823, 0.827)	0.510 (0.506, 0.514)	0.738 (0.736, 0.74)	0.752 (0.75, 0.754)
XGBoost	0.826 (0.824, 0.828)	0.513 (0.509, 0.517)	0.749 (0.747, 0.751)	0.751 (0.749, 0.753)
LightGBM	0.819 (0.817, 0.821)	0.517 (0.512, 0.522)	0.749 (0.746, 0.752)	0.751 (0.748, 0.754)
Decision Tree	0.648 (0.647, 0.649)	0.277 (0.275, 0.279)	0.306 (0.305, 0.307)	0.804 (0.803, 0.805)
LR	0.695 (0.693, 0.697)	0.299 (0.295, 0.303)	0.633 (0.631, 0.635)	0.636 (0.634, 0.638)
SVM	0.795 (0.793, 0.797)	0.472 (0.468, 0.476)	0.719 (0.716, 0.722)	0.727 (0.725, 0.729)
MLP	0.774 (0.772, 0.776)	0.442 (0.437, 0.447)	0.711 (0.708, 0.714)	0.714 (0.711, 0.717)

To elucidate the features that influenced this prediction model further, we adopted the SHAP summary value of RF. This figure shows if the features were strongly connected to EGFR mutation. In other words, the higher the SHAP value, the greater possibility of EGFR mutation. As shown in the picture, smoking consumption, gender, cholesterol, age, and albumin globulin ratio ranked as the top five among all variables. The SHAP dependence plot can also be used to explain how a single factor affects the result of the model. The y-axis shows the SHAP value of single feature, and the value of different features is showed in the x-axis. We could vividly describe the tendency of each feature with the changing plots. SHAP value for specific features exceeding zero represents an increased risk of incidence of EGFR mutation.

## Discussion

Lung cancer is traditionally divided into two broad histologic categories: non–small cell lung cancer (NSCLC) and small cell lung cancer (SCLC). NSCLC represents more than 80% to 85% of lung cancers, of which approximately 40% are adenocarcinoma (11). In a development of precision medicine, we have found the important influence of gene mutation in oncotherapy and prognosis. EGFR mutation is one of the most common genotypes of lung cancer and occurs in at least 50% of NSCLC in Asia (17). TKIs show marked clinical benefits in EGFR mutation patients, compared with the conventional chemical therapy (18). In clinical work, gene examination requires biopsy and sequence testing, which cannot be detected in some cases, because of insufficient samples, the financial situation of patients, and so on. This impedes patients’ therapy and prognosis in some degree. Moreover, obtaining samples usually depends on invasive methods, which have risk of bleeding, excessive damage, and so on. Hence, non-invasive method to predict EGFR mutation is pressing. In the present prediction models, the LR is commonly used, with the AUC ranging from 0.6 to 0.8 (12, 19).

**Figure 2:**
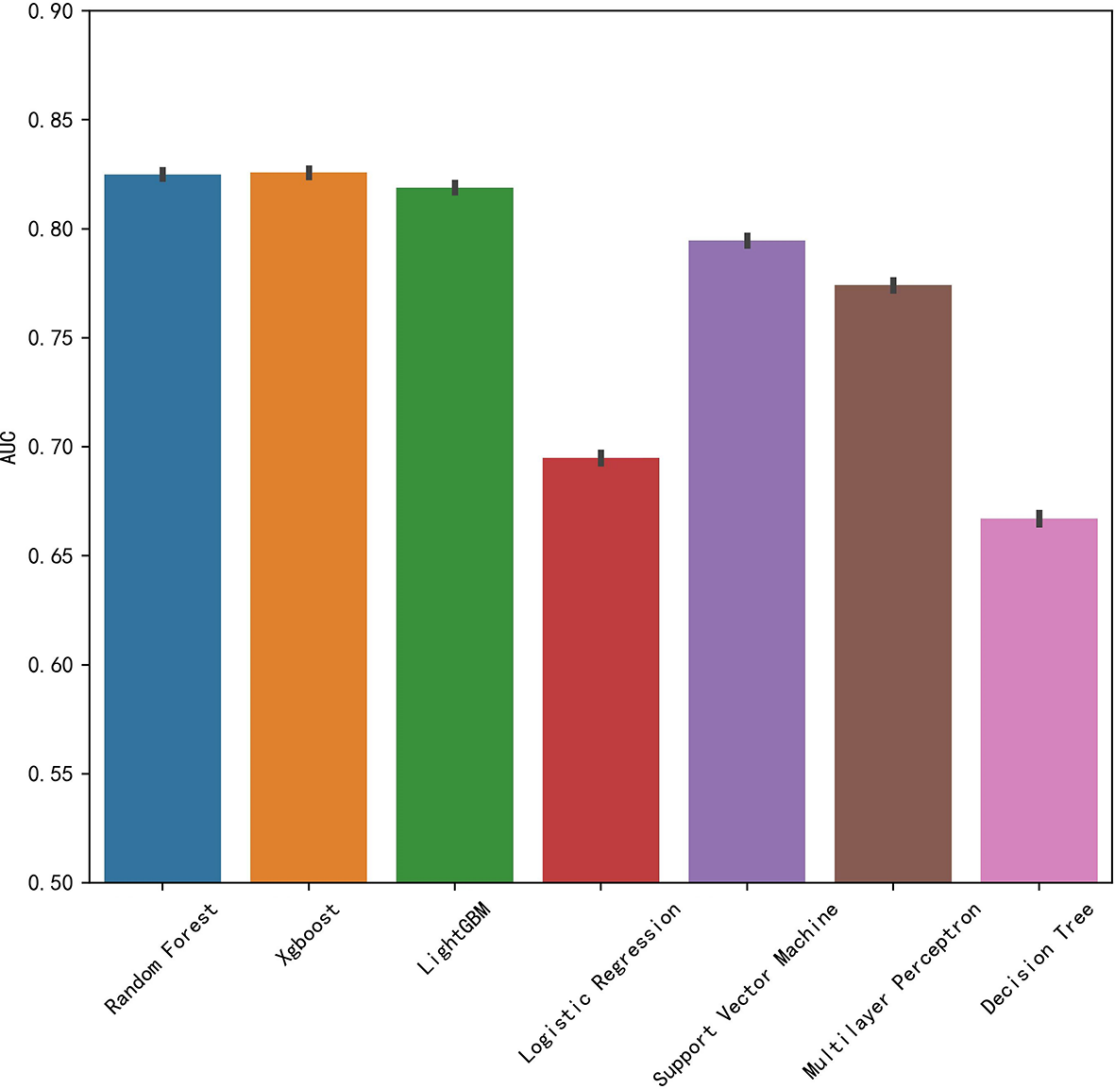
Comparison of AUCs among seven machine learning models with bar graph.

In this retrospective study, we included more advanced MLAs using 91 features and filtrated models by LASSO algorithm and clinical experience. It indicated that RF gained better performance (**Figure 1**). To describe visually, we used a bar graph (**Figure 2**) to interpret the discrepancy between the traditional and novel machine learning methods, and we found that RF, XGBoost, and light GBM were superior compared with the traditional ones, like decision tree, multilayer perceptron, and SVM. The RF model got the best performance, whereas the LR model got the worst.

**Figure 3:**
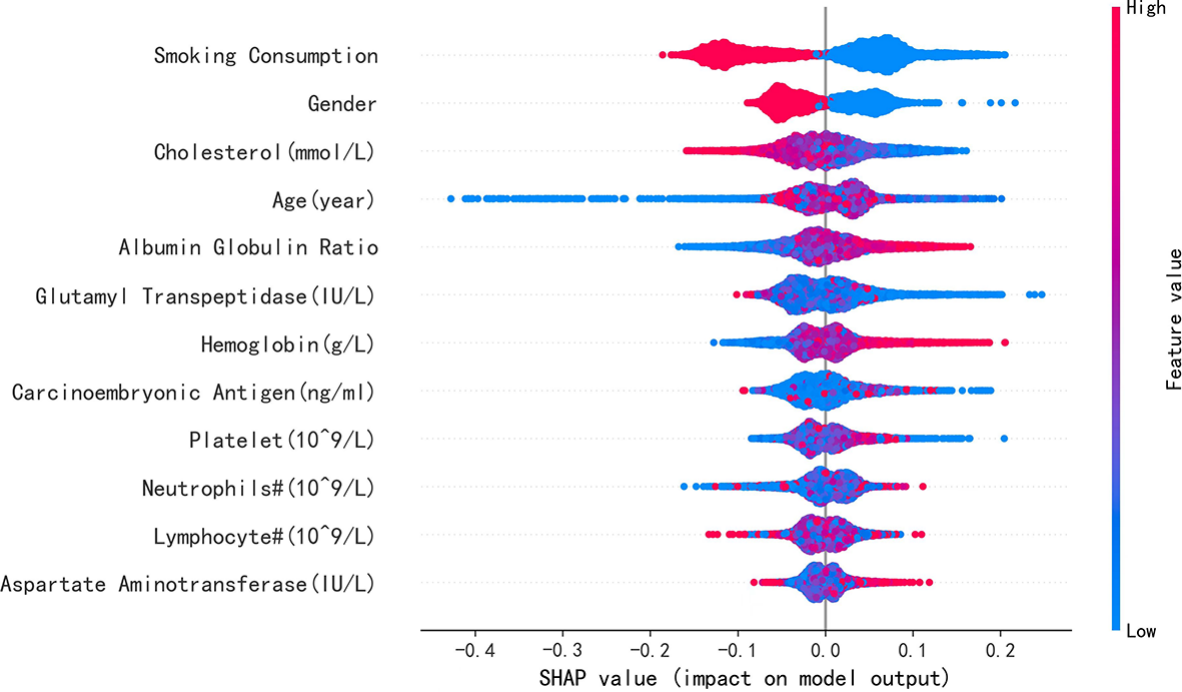
SHAP summary plot of the 12 features of the RF model. The higher the SHAP value of single feature, the higher the possibility of EGFR mutation. Red represents closer with this mutation, and blue represents apposite possibility.

**Figure 4:**
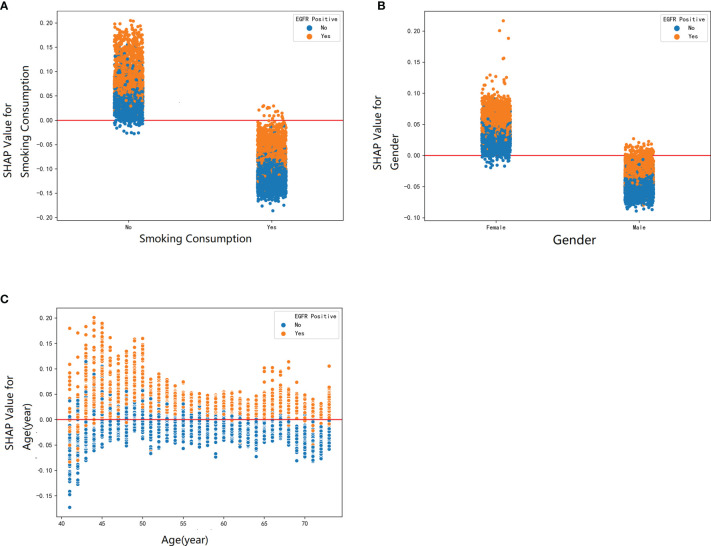
The SHAP dependence plot was used to explain how a single factor affects the result in this RF model. SHAP value for specific feature exceeding zero represents an increased risk of incidence of EGFR mutation. The demographic factors: **(A)** smoking consumption, **(B)** gender, and **(C)** age.

**Figure 5:**
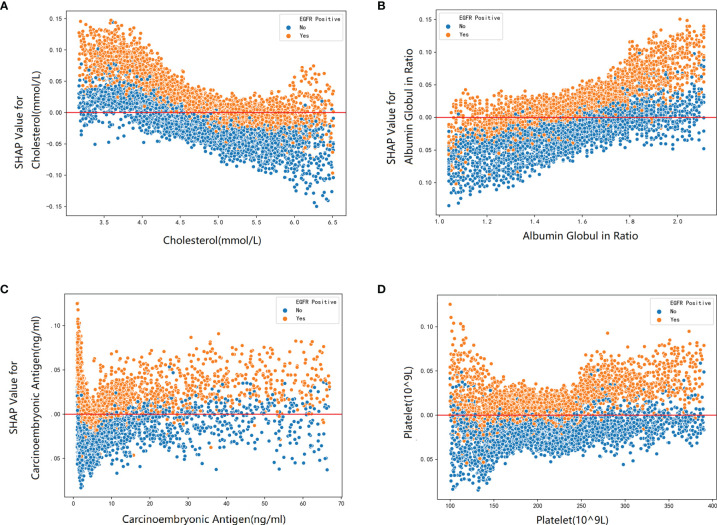
The SHAP dependence plot about blood markers: **(A)** cholesterol, **(B)** albumin globulin ratio, **(C)** carcinoembryonic, and **(D)** platelet.

Furthermore, our SHAP summary plot (**Figure 3**) provided an explicable machine learning model to predict EGFR mutation in LA with large scales of samples and clinical features. Smoking consumption, sex, cholesterol, age, and albumin globulin ratio were in the top five variables related to EGFR mutation. From the previous epidemiological studies, it has been found that non-smoking characteristics among female patients are non-negligible (18, 20–23); consistent with our study, female patients who are non-smokers were more likely to have EGFR mutation (**Figures 4A, B**). Interestingly, we found that people under 40 years old were not prone to this mutation, but with age, the trend of change has no significant characteristics (**Figure 4C**). The possible reason is that there are more co-influential factors such as aging, so the independent age change cannot exhibit a specific effect. As for metabolic indicators, mutation risk is elevated for participants with lower cholesterol and higher albumin globulin ratio (**Figures 5A, B**). The increased albumin globulin ratio might be explained by suppressed immune function or high and aberrant expression of normal proteins (24), which were called tumor-associated antigens. The relationship and the specific mechanism need further studies. Increased CEA (25, 26), which is often observed in LA, was also included. We found that CEA (**Figure 5C**) was associated with the mutation but we also easily saw that there was no significant connection with the change, similar to glutamyl transpeptidase, neutrophils, and lymphocytes. As for platelet, we could also see that patients with platelets exceeding the normal range were close to EGFR mutation (**Figure 5D**). Lots of research had provided evidence of venous thromboembolism incidence in EGFR mutation patients and lead to an inconsistent conclusion (27–29). In our study, the result supported the positive change. Differences between genotypes and therapy choices need further study.

Despite the encouraging performance of our model, this study also has several limitations. First, although the sample size was relatively large in our retrospective study, we only examined patients in West China Hospital; moreover, it presented partial information about LA in the Western region. Hence, there was indeed bias in this study, and further multicenter and prospective study in future is needed. Second, we did not include the imaging statistics in our predicting model. Third, only EGFR mutation was included in our study; in future work, the relationship between EGFR and other genetic mutations, like c-ros oncogene 1 (ROS-1), ALK, and Kirsten rat sarcoma viral oncogene (KRAS), or different subtypes of EGFR, can be explored.

## Conclusion

In brief, accurate and rapid EGFR discrimination is valuable for patients, both resectable and inaccessible to surgery. Traditional diagnosis methods are limited to a large proportion of patients. We established the explainable machine model to predict EGFR mutation in patients with LA, which can be referred to in the clinical diagnosis as a non-invasive method, which may guide or assist treatment of patients who lack pathologic diagnosis.

## Data Availability Statement

The original contributions presented in the study are included in the article/**Supplementary Material**. Further inquiries can be directed to the corresponding author.

## Ethics Statement

The studies involving human participants were reviewed and approved by the Ethics Committee on Biomedical Research, West China Hospital of Sichuan University. The ethics committee waived the requirement of written informed consent for participation.

## Author Contributions

WL devised concept of article. RY wrote the manuscript. XX and HW processed the data. XX and WL reviewed the article. All authors contributed to the article and approved the submitted version.

## Funding

This work was supported by National Natural Science Foundation of China (Nos. 92159302, 81871890, and 91859203 to WL).

## Conflict of Interest

The authors declare that the research was conducted in the absence of any commercial or financial relationships that could be construed as a potential conflict of interest.

## Publisher’s Note

All claims expressed in this article are solely those of the authors and do not necessarily represent those of their affiliated organizations, or those of the publisher, the editors and the reviewers. Any product that may be evaluated in this article, or claim that may be made by its manufacturer, is not guaranteed or endorsed by the publisher.
