# Supplementary material for: Explainable Machine Learning Model to Prediction EGFR Mutation in Lung Cancer
Source: Front Oncol. 2022 Jun 23;12:924144. doi: 10.3389/fonc.2022.924144 (PMC9259982; doi:10.3389/fonc.2022.924144)
Supplement: Supplementary file 1 [file Table_1.docx]

Supplementary Material

| **Table S1** Patient characteristics and blood markers | | | | |
| --- | --- | --- | --- | --- |
| **Variables** |  | **EGFR-wild type** | **EGFR-mutation** | **P-Value** |
| **Patient population, n (n%)** |  | 3886 | 3527 |  |
| **Demographic data** |  |  |  |  |
| Gender, n (%) |  |  |  | <0.001 |
| Female |  | 1446 (37.210) | 1970 (55.855) |  |
| Male |  | 2440 (62.790) | 1557 (44.145) |  |
| Age(year), mean (SD) |  | 56.965 (10.749) | 56.893 (10.197) | 0.768 |
| Smoking Consumption, n (%) |  |  |  | <0.001 |
| No |  | 1814 (46.873) | 2490 (71.001) |  |
| Yes |  | 2056 (53.127) | 1017 (28.999) |  |
| Smoking Time(year), mean (SD) |  | 30.877 (10.255) | 26.209 (11.035) | <0.001 |
| Quit Smoking, n (%) |  |  |  | 0.001 |
| No |  | 405 (18.056) | 292 (22.956) |  |
| Yes |  | 1838 (81.944) | 980 (77.044) |  |
| Alcoholism, n (%) |  |  |  | <0.001 |
| Never |  | 2413 (62.708) | 2633 (75.531) |  |
| Occasion |  | 760 (19.751) | 483 (13.855) |  |
| Often |  | 675 (17.542) | 370 (10.614) |  |
| Drinking Time(year), mean (SD) |  | 28.855 (10.614) | 25.535 (11.667) | <0.001 |
| Daily Smoking, mean (SD) |  | 19.057 (5.688) | 17.780 (6.341) | <0.001 |
| Alcohol Consumption(year), mean (SD) |  | 173.462 (138.643) | 145.190 (113.293) | <0.001 |
| Smoking Cessation Time(year), median [Q1,Q3] |  | 2.000 [1.000,7.000] | 2.000 [1.000,8.000] | 0.002 |
| Temperature (℃), mean (SD) |  | 36.466 (0.215) | 36.467 (0.209) | 0.791 |
| Pulse(cpm), mean (SD) |  | 85.076 (11.137) | 84.469 (10.957) | 0.018 |
| Breathing(cpm), mean (SD) |  | 19.696 (0.858) | 19.678 (0.833) | 0.353 |
| Systolic pressure(mmHg), mean (SD) |  | 121.922 (12.757) | 122.664 (13.780) | 0.017 |
| Diastolic pressure(mmHg), mean (SD) |  | 76.640 (8.582) | 77.199 (9.167) | 0.007 |
| Weight(kg), mean (SD) |  | 61.170 (9.398) | 59.232 (9.707) | <0.001 |
| Height(cm), mean (SD) |  | 163.432 (7.593) | 161.268 (7.699) | <0.001 |
| Cough, n (%) |  |  |  | <0.001 |
| No |  | 2721 (70.021) | 2730 (77.403) |  |
| Yes |  | 1165 (29.979) | 797 (22.597) |  |
| Expectoration, n (%) |  |  |  | <0.001 |
| No |  | 3262 (83.942) | 3146 (89.198) |  |
| Yes |  | 624 (16.058) | 381 (10.802) |  |
| Dyspnea, n (%) |  |  |  | 0.103 |
| No |  | 3526 (90.736) | 3239 (91.834) |  |
| Yes |  | 360 (9.264) | 288 (8.166) |  |
| Fever, n (%) |  |  |  | 0.142 |
| No |  | 3857 (99.254) | 3511 (99.546) |  |
| Yes |  | 29 (0.746) | 16 (0.454) |  |
| Hemoptysis, n (%) |  |  |  | 0.528 |
| No |  | 3700 (95.214) | 3346 (94.868) |  |
| Yes |  | 186 (4.786) | 181 (5.132) |  |
| Chest Pain, n (%) |  |  |  | 0.161 |
| No |  | 3686 (94.853) | 3371 (95.577) |  |
| Yes |  | 200 (5.147) | 156 (4.423) |  |
| Night Sweat, n (%) |  |  |  | 0.128 |
| No |  | 3880 (99.846) | 3526 (99.972) |  |
| Yes |  | 6 (0.154) | 1 (0.028) |  |
| Hoarseness, n (%) |  |  |  | <0.001 |
| No |  | 3834 (98.662) | 3509 (99.490) |  |
| Yes |  | 52 (1.338) | 18 (0.510) |  |
| Chills, n (%) |  |  |  | 0.024 |
| No |  | 3886 (100.000) | 3522 (99.858) |  |
| Yes |  | 0(0.000) | 5 (0.142) |  |
| **Blood markers** |  |  |  |  |
| RBC (*10^12/L), mean (SD) |  | 4.055 (0.626) | 4.150 (0.621) | <0.001 |
| HB (g/L), mean (SD) |  | 120.959 (17.833) | 122.760 (17.470) | <0.001 |
| Hematocrit(%), mean (SD) |  | 0.374 (0.052) | 0.379 (0.050) | <0.001 |
| MCV (fL), mean (SD) |  | 92.828 (6.214) | 92.058 (6.080) | <0.001 |
| MCH (pg), mean (SD) |  | 30.059 (2.208) | 29.863 (2.195) | <0.001 |
| MCHC (g/L), mean (SD) |  | 323.318 (11.483) | 323.840 (11.740) | 0.062 |
| RDW-CV (fL), mean (SD) |  | 14.858 (1.806) | 14.649 (1.778) | <0.001 |
| RBC Distribution Width SD(fL), mean (SD) |  | 49.837 (6.458) | 48.683 (6.153) | <0.001 |
| Platelet(10^9/L), mean (SD) |  | 215.069 (83.880) | 211.581 (81.704) | 0.079 |
| Leukocyte Count(10^9/L), mean (SD) |  | 6.130 (2.438) | 5.923 (2.278) | <0.001 |
| Neutrophils%, mean (SD) |  | 64.071 (12.673) | 63.993 (11.888) | 0.794 |
| Lymphocyte%, mean (SD) |  | 24.315 (10.758) | 24.825 (10.108) | 0.043 |
| Monocytes%, mean (SD) |  | 8.151 (3.096) | 7.829 (2.967) | <0.001 |
| Eosinophil%, mean (SD) |  | 2.235 (1.829) | 2.368 (1.891) | 0.003 |
| Basophil%, mean (SD) |  | 0.480 (0.328) | 0.466 (0.330) | 0.090 |
| Neutrophils(10^9/L), mean (SD) |  | 4.101 (2.129) | 3.944 (1.991) | 0.001 |
| Lymphocyte(10^9/L), mean (SD) |  | 1.373 (0.550) | 1.362 (0.527) | 0.382 |
| Monocytes(10^9/L), mean (SD) |  | 0.484 (0.218) | 0.447 (0.203) | <0.001 |
| Eosinophil(10^9/L), mean (SD) |  | 0.126 (0.107) | 0.132 (0.109) | 0.034 |
| Basophil(10^9/L), mean (SD) |  | 0.029 (0.020) | 0.027 (0.020) | <0.001 |
| Immature Granulocyte, mean (SD) |  | 0.032 (0.027) | 0.027 (0.025) | <0.001 |
| Immature Granulocyte(%), mean (SD) |  | 0.495 (0.345) | 0.429 (0.328) | <0.001 |
| TBil (umol/L), mean (SD) |  | 9.415 (3.710) | 9.851 (3.869) | <0.001 |
| DBil (umol/L), mean (SD) |  | 2.943 (1.241) | 3.076 (1.297) | <0.001 |
| ALT (IU/L), mean (SD) |  | 23.867 (12.185) | 23.010 (12.197) | 0.003 |
| Indirect Bilirubin (umol/L), mean (SD) |  | 6.437 (2.707) | 6.745 (2.810) | <0.001 |
| Total Protein (g/L), mean (SD) |  | 67.937 (5.992) | 67.392 (6.075) | <0.001 |
| Albumin (g/L), mean (SD) |  | 40.553 (4.500) | 40.904 (4.473) | 0.001 |
| Globulin (g/L), mean (SD) |  | 27.376 (4.642) | 26.515 (4.365) | <0.001 |
| Albumin Globulin Ratio, mean (SD) |  | 1.527 (0.329) | 1.587 (0.320) | <0.001 |
| Creatinine (umol/L), mean (SD) |  | 64.468 (15.338) | 62.873 (15.923) | <0.001 |
| UA (umol/L), mean (SD) |  | 317.929 (87.196) | 295.164 (89.404) | <0.001 |
| AST/ALT, mean (SD) |  | 1.177 (0.426) | 1.232 (0.446) | <0.001 |
| Glucose(mmol/L), mean (SD) |  | 5.218 (0.799) | 5.177 (0.769) | 0.026 |
| AST(IU/L), mean (SD) |  | 24.854 (8.265) | 24.952 (8.444) | 0.623 |
| ALP(IU/L), mean (SD) |  | 87.852 (28.025) | 85.793 (29.276) | 0.003 |
| Glutamyl Transpeptidase (IU/L), mean (SD) |  | 36.925 (22.541) | 33.808 (22.600) | <0.001 |
| Triglyceride (mmol/L), mean (SD) |  | 1.501 (0.667) | 1.473 (0.656) | 0.077 |
| BUN (mmol/L), mean (SD) |  | 5.031 (1.545) | 4.998 (1.561) | 0.368 |
| Cholesterol (mmol/L), mean (SD) |  | 4.773 (1.006) | 4.726 (1.002) | 0.053 |
| Calcium (mmol/L), mean (SD) |  | 2.230 (0.124) | 2.225 (0.128) | 0.062 |
| Magnesium (mmol/L), mean (SD) |  | 0.850 (0.088) | 0.857 (0.085) | <0.001 |
| Phosphorus (mmol/L), mean (SD) |  | 1.120 (0.185) | 1.131 (0.191) | 0.019 |
| HDL (mmol/L), mean (SD) |  | 1.261 (0.365) | 1.328 (0.366) | <0.001 |
| LDL (mmol/L), mean (SD) |  | 2.808 (0.807) | 2.754 (0.814) | 0.005 |
| Sodium (mmol/L), mean (SD) |  | 140.999 (2.710) | 141.094 (2.602) | 0.135 |
| Potassium (mmol/L), mean (SD) |  | 4.107 (0.361) | 4.098 (0.363) | 0.290 |
| Chlorine (mmol/L), mean (SD) |  | 102.047 (3.390) | 102.227 (3.398) | 0.026 |
| CO2CP (mmol/L), mean (SD) |  | 25.057 (2.834) | 25.161 (2.779) | 0.124 |
| Anion Gap (mg/L), mean (SD) |  | 18.038 (3.075) | 17.903 (2.942) | 0.060 |
| Cystatin C (mg/L), mean (SD) |  | 0.964 (0.168) | 0.939 (0.173) | <0.001 |
| Hydroxybutyric Acid (mmol/L), mean (SD) |  | 0.099 (0.041) | 0.103 (0.043) | <0.001 |
| PT (s), mean (SD) |  | 11.587 (1.067) | 11.458 (1.011) | <0.001 |
| INR, mean (SD) |  | 0.994 (0.093) | 0.985 (0.088) | <0.001 |
| APTT (s), mean (SD) |  | 28.151 (3.866) | 27.947 (3.606) | 0.029 |
| APTT Ratio, mean (SD) |  | 1.017 (0.136) | 1.012 (0.129) | 0.158 |
| TT (s), mean (SD) |  | 18.298 (1.225) | 18.236 (1.193) | 0.040 |
| TT Ratio, mean (SD) |  | 1.123 (0.075) | 1.118 (0.074) | 0.012 |
| FIB (g/L), mean (SD) |  | 3.730 (1.208) | 3.508 (1.117) | <0.001 |
| AT-III (%), mean (SD) |  | 89.898 (13.127) | 92.110 (13.037) | <0.001 |
| FDP (mg/L), mean (SD) |  | 4.158 (2.499) | 4.144 (2.542) | 0.856 |
| D-Dimer (mg/I FEU), mean (SD) |  | 1.320 (1.208) | 1.296 (1.234) | 0.501 |
| CEA (ng/ml), median [Q1,Q3] |  | 6.120 [2.440,25.348] | 6.640 [2.300,30.797] | 0.881 |
| Non Small Cell Lung Cancer Antigen (ng/ml), mean (SD) |  | 4.650 (3.290) | 4.236 (3.175) | <0.001 |
| NSE (ng/ml), mean (SD) |  | 13.742 (4.598) | 13.962 (4.753) | 0.065 |
| **TNM stage** |  |  |  |  |
| T, n (%) |  |  |  | <0.001 |
| T0 |  | 56 (1.525) | 25 (0.762) |  |
| T1 |  | 496 (13.508) | 487 (14.848) |  |
| T2 |  | 1242 (33.824) | 1317 (40.152) |  |
| T3 |  | 509 (13.862) | 456 (13.902) |  |
| T4 |  | 1258 (34.259) | 979 (29.848) |  |
| Tx |  | 111 (3.023) | 16 (0.488) |  |
| N, n (%) |  |  |  | <0.001 |
| N0 |  | 769 (20.948) | 870 (26.516) |  |
| N1 |  | 420 (11.441) | 364 (11.094) |  |
| N2 |  | 1119 (30.482) | 1215 (37.031) |  |
| N3 |  | 1268 (34.541) | 745 (22.706) |  |
| Nx |  | 95 (2.588) | 87 (2.652) |  |
| M, n (%) |  |  |  | <0.001 |
| M0 |  | 1352 (44.977) | 1228 (46.375) |  |
| M1a |  | 626 (20.825) | 473 (17.863) |  |
| M1b |  | 524 (17.432) | 444 (16.767) |  |
| M1c |  | 431 (14.338) | 461 (17.409) |  |
| Mx |  | 73 (2.428) | 42 (1.586) |  |
| Stages, n (%) |  |  |  | <0.001 |
| Ⅰ |  | 1129 (31.563) | 1103 (33.938) |  |
| Ⅱ |  | 29 (0.811) | 35 (1.077) |  |
| Ⅲ |  | 152 (4.249) | 71 (2.185) |  |
| Ⅳ |  | 2267 (63.377) | 2041 (62.800) |  |

**
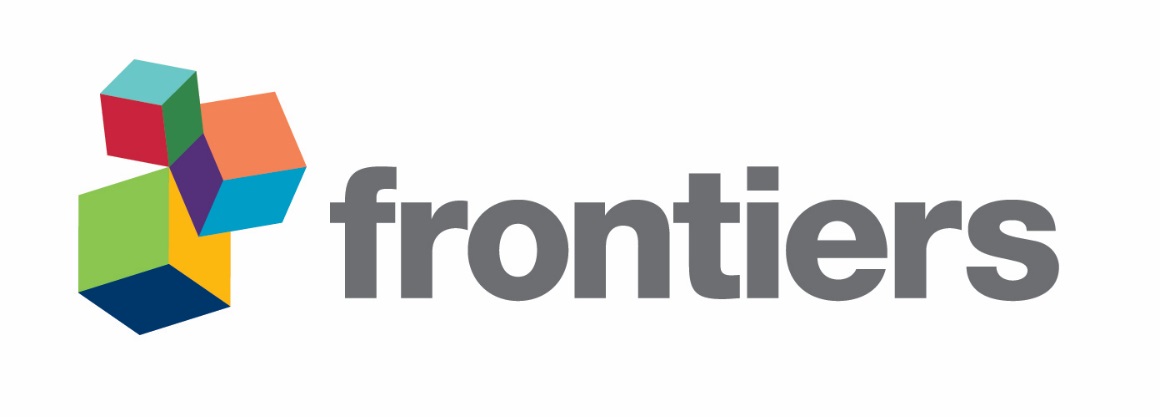
**
